# Case-control study on analgesics and nephropathy (SAN): protocol

**DOI:** 10.1186/1471-2369-6-9

**Published:** 2005-08-08

**Authors:** Lothar AJ Heinemann, Edeltraut Garbe, Michael Lewis, Fokko van der Woude, Helmut Graf

**Affiliations:** 1 Centre for Epidemiology & Health Research Berlin, Invalidenstr. 115, 10115 Berlin, Germany; 2 Institute for Clinical Pharmacology, Charité-University Medicine Berlin, Schumannstr. 20–21, 10117 Berlin, Germany; 3 EPES Epidemiology, Pharmacoepidemiology and Systems Research GmbH, Wulfstr. 8, 12165 Berlin, Germany; 4 Nephrology, 5. Med. Klinik, Klinikum Heidelberg-Mannheim, Theodor-Kutzer-Ufer 1–3, 68167 Mannheim, Germany; 5 Neprologie, Krankenanstalt der Stadt Wien, Rudolfstifting, 3. Med. Abteilung, Juchgasse 25, 1030 Wien, Austria

## Abstract

**Background:**

The association between intake of non-phenacetin-containing analgesics and the occurrence of chronic renal failure is still controversially discussed. A new epidemiologic study was planned and conducted in Germany and Austria.

**Methods/design:**

The objective of the international, multicenter case-control study was to evaluate the association between end-stage renal disease (ESRD) and use of non-phenacetin-containing analgesics with particular emphasis on combined formulations. A targeted sample of 1000 new (incident) dialysis patients, aged less than 50 years, was planned to recruit between January 1, 2001 and December 31, 2004. The age limit was chosen to avoid contamination of the study population with phenacetin-containing analgesics to the extent possible. Four control subjects per ESRD case, matched by age, sex, and region were selected from the population living in the region the case came from.

Lifetime exposure to analgesics and potential renal risk factors were recorded in a single face-to-face interview. A set of aids was introduced to reinforce the memory of study participants.

A standardized, pre-tested interview questionnaire (participants), a medical documentation sheet (physicians in dialysis centres), a logbook for all activities (dialysis centres) were used to collect the necessary data.

Quality management consisted of the standardized procedures, (re-) training and supervision of interviewers, regular checks of all incoming data for completeness and plausibility.

The study is scientifically independent and governed by a international Scientific Advisory Committee that bridged the gap between the sponsoring companies and the investigators. Also other advisory groups assisted the managing committee of the study. All relevant German and Austrian nephrological associations supported the study, and the study design was carefully reviewed and approved by the Kidney Foundation of Germany.

**Discussion:**

The study is expected to answer the main research question by end 2005. There is however a high potential for various biases that we tried to address with adequate measure. One limitation however cannot be overcome: The methodologically needed age-limitation of the study will make it not easy to generalize the results to age groups over 50 years. It might be suggested to repeat the study for persons over 50 years in 10 years when contamination with phenacetin use early in life is likely to be outgrown.

## Background

It was long time ago that Dubach et al. published their paper on phenacetin nephropathy [1]. At that time, phenacetin-containing analgesics were freely available in bulk to all workers in the Swiss watch industry [1], and sachets of phenacetin-containing analgesic powders were even given as a tip for small services to apprentices in the Huskvarna factory in Sweden [2]. Several epidemiological studies were undertaken to clarify the association between phenacetin overuse and development of end stage renal disease (ESRD)^1 ^(see [3,4] for review). As a result, phenacetin was banned at different times in almost all states of the world. However, although "phenacetin nephropathy" – thought to develop only after decades of chronic phenacetin overuse – could not be expected to disappear soon, nephrologists suspected that non-phenacetin analgesic combinations, especially those containing paracetamol and aspirin (plus caffeine) might cause ESRD as well and introduced the hypothetical term "analgesic nephropathy". Since empirical data were scarce, this hypothesis engendered some controversy. Safety concerns led the regulatory authorities of Germany, Austria and Switzerland to initiate a scientific re-evaluation. A peer review committee of scientists was jointly selected by the regulatory authorities and the pharmaceutical industry and was asked to critically review data on the relationship between non-phenacetin combined analgesics and nephropathy. *The committee's two main conclusions were that (1) there is insufficient evidence to associate non-phenacetin combined analgesics with nephropathy and that (2) new studies should be done to provide appropriate data to resolve the question *[5]. Meanwhile the US Food and Drug Administration (FDA) had approved an analgesic combination containing paracetamol, aspirin, and caffeine as safe and effective for the use in uncomplicated migraine [6].

The Scientific Advisory Committee recommended the new study to be conducted within a relatively short time period to meet regulatory needs. The Drug Authorities (Germany, Austria, Switzerland) agreed that the year 2006 is as an acceptable deadline for obtaining final results. The discussions emphasized that the study should be designed and conducted in a way that ruled out any possibility of contamination by prior phenacetin use. In addition, it was agreed to conduct the study in those countries that had expressed concern about the problem, since the relevant compound analgesics are available on the markets of these countries and have a sufficient market share.

A further question to be addressed was whether caffeine co-formulated with analgesics could lead to a dependence on analgesics, contribute to heavy long-term use, and thereby result in a higher risk of ESRD. Although an additional Expert Meeting on Caffeine found no evidence for such an effect [7], and although a further special review did not reveal any substantial data to support a pivotal role of caffeine [8], the new study was intended to provide information on the role of caffeine in risk development.

Since ESRD is a rare outcome, a case-control study seemed to be the appropriate design (initially called "Study on Analgesic Nephropathy", SAN). Neither a study outside Europe (where combined analgesics are used to a lesser extent) nor long-term prospective cohort studies were regarded as acceptable alternatives, since the latter would not provide timely results. It was not possible to conduct a historical cohort study, because the required data were not available.

The protocol of the case control study as agreed upon by the Scientific Advisory Committee was approved by the regulatory authorities of Germany, Austria and Switzerland and was also reviewed by 'The Lancet' without any recommendation for change [personal communication; Prof. Fox, 2004] The initial study protocol can be read in the website ).

The objective of this publication is to present the study protocol with some minor revisions as approved by the Scientific Advisory Committee prior to the final analyses and subsequent publication in 2006.

## Methods/design

### General study design and rationale

A case-control study design was chosen because the study addresses the potential association between a rare medical condition (ESRD) and a relatively common exposure (analgesics) and because it needed to be conducted within a short period of time at affordable costs. Cases would be individuals initiating dialysis (incident ESRD) at one of the participating centers, and controls would be taken from the general population.

Two major concerns influenced the planning of the study:

- To exclude the possibility of inadvertent contamination with phenacetin among study subjects, study participation was restricted to individuals under 50 years of age. While this measure greatly impedes case accrual (only 25% of incident ESRD is within this age range), it assures minimal phenacetin contamination, because phenacetin use declined in the 1970ies and the drug was removed from the market in the countries under study in mid to late 1980. This age limit assures good recall of drug histories among these young study subjects and captures the group most likely to be affected if the metabolite of phenacetin, paracetamol, were associated with ESRD.

- The second issue concerns the time from onset of the disease until the diagnosis of ESRD which leads to dialysis, which is estimated to be between 5 to 10 years. Because the applicability of this pattern in association with a putative cause such as analgesic use is unclear, the protocol permits analyses of lag time and of appropriate subgroups.

### Objectives of the study

The study was designed to address as many questions as possible about the association between ESRD and analgesic use in the range of recommended dosage with sufficient statistical power and in a timely manner.

The primary study objective is to determine whether a significant association between the occurrence of ESRD and the use of phenacetin-free analgesics in the average population exists or not.

A. The following three groups are of primary interest in the context of the main research objectives (basis for sample size estimations):

- Use of any phenacetin-free analgesics

- Use of any phenacetin-free analgesics in fixed combinations ("combis")

- Use of any phenacetin-free single substance – analgesics ("monos")

B. Drug-specific analyses will be done for groups with sufficient numbers, focusing on paracetamol with and without other analgesics, and with and without caffeine in the formulation.

C. In addition to the comparison between use and non-use (see "exposure"), the magnitude of use, such as dose, duration, cumulative lifetime dose per duration of use and other measures for the extent of exposure will be assessed. The desirable sub-group analyses (cf. exposure groups) are listed in advance for transparency, although it is recognized that some analyses will lack statistical power.

If a significant association of analgesic use and risk of ESRD becomes apparent, the secondary objective of the study is to analyze the patterns of use among individuals with excessive analgesics use (over 30% in excess of the recommended dose) and to address the role of caffeine co-formulated with analgesics and in conjunction with caffeine use in beverages, medical conditions, and indicators of general health behavior. This, however, is not part of the main research question focused on the risk of ESRD in the average population, i.e. not in specific sub-groups.

### Study design

The study is conducted as a multicenter case-control study in Germany and Austria. New (incident) cases of ESRD will be collected from dialysis centers. On average, four population controls will be individually matched per case for age, gender and region the case came from.

Cases are defined as persons with end-stage renal disease (ESRD), newly admitted to a chronic dialysis program. Since there may be a considerable lag time between the underlying condition and the diagnosis of ESRD and because the underlying renal disease itself may have led to the use of analgesics, four index dates preceding the actual diagnosis of ESRD were defined. All potentially associated exposures (analgesic use, exposure to other chemicals at the work site), and conditions or complaints occurring after a given index date will not be considered in the statistical analyses. The four index dates were defined as follows:

• *Index date 1*: Time of first entry into the dialysis program for cases; time of personal interview for controls.

• *Index date *2: Time the case was first informed about incipient renal failure from the treating physician; the index date for the control is the index date of the respective case.

• *Index date 3 *represents an arbitrarily defined lag-time of 5 years prior to the start of dialysis; the index date for the control is the index date of the respective case.

• *Index date 4 *represents an arbitrarily defined lag-time of 10 years prior to the start of dialysis; the index date for the control is the index date of the respective case.

Since index date 2 might be subject to information- and recall bias, index date 3 is considered to be the most reliable index date for risk estimates. Index 2 might be used if the number of analgesic users is too small at index date 3. The analgesic exposure of cases and controls at index date 4 (10 years before terminal renal insufficiency) was predicted to become too small for stable analyses, although this date might be biologically sound. The figure shows the relationship between index dates and putative analgesic use. Note that Index date 2 may have a wide range.

### Outcome measure

The outcome measure is the relative risk of ESRD associated with use of analgesics either as single or combined formulation: heavy use (see later) vs. no use; lifetime dose, or duration of use.

### Study participants

#### Recruitment of centers

The study aimed to collaborate with as many dialysis centers as possible in Germany and Austria to guarantee a sufficiently high and complete recruitment of incident ESRD patients in this young age group. A compensation for the time spent was offered to controls – if requested- and on demand to cases (up to 20 €) as well as to dialysis centers (up to 40€ for completing the medical history and diagnostic documentation).

The Clinical Principal Investigators, Prof. van der Woude for Germany and Prof. Graf for Austria, were responsible for organizing the recruitment of dialysis centers via the relevant professional organizations. In Germany, a joint effort of three nephrological associations under the auspices of the German Kidney Foundation facilitated contacts to hundreds of dialysis centers and thereby a good study performance. The German Kidney Foundation independently approved the study protocol, established an organizational network for the smooth collaboration among centers and with the Data Management and Coordination Center (DMCC – ZEG Berlin), and officially invites the dialysis units on behalf of the German Kidney Foundation. In Austria, Prof. Graf organized the study after approval from the Nephrological Association of Austria, which officially invited the dialysis centers' participation. In both countries, an information and training program was introduced for all dialysis centers participating in the study.

By the end of 2004, 259 dialysis centers had agreed to participate in the study and 170 contributed at least one incident case of ESRD. Details about centers and geographical distribution can be seen at the website of the SAN study .

#### Recruitment of cases and controls

All study participants received a comprehensive letter of invitation which explained the study objective of investigating various health outcomes with respect to life-time exposure to chemicals including drug treatment. No reference to analgesics is made in the invitation letter. The letter in addition provides some sample questions about exposure to chemicals and health outcomes similar to those that might be asked in the study interview. One intention of the letter is to stimulate controls, prior to the interview, to carefully think about their lifetime history of exposure to any chemicals, including drugs, in relation to medical and other conditions. This approach was chosen to minimize recall bias that could arise because cases in the past were likely asked more often for potential exposures than controls. Cases may therefore be more fully aware of previous exposures than controls. The letter was therefore designed to create a more similar interview situation for cases and controls by stimulating controls to reflect about prior exposures and health outcomes before the study interview.

#### Cases

Cases are persons with terminal renal failure with a first admission to renal replacement treatment because of severe renal insufficiency – end stage renal disease ("ESRD cases"). Individuals with conditions leading to an *acute *dialysis program were not eligible as cases. The renal function of subjects included as cases was re-evaluated after 3 months. Patients who were found to be acute dialysis patients or patients for re-dialysis after transplant rejection were then excluded.

Logbooks were kept in all participating centers for the duration of their participation in the study to monitor case accrual and completeness of ESRD patient capture. It was anticipated that not all centers would be able to participate for the entire study period, some entering late, and others discontinuing for various reasons – e.g. due to excessive workload.

Cases are eligible if they are new (incident chronic dialysis, not acute or recurrent) and under 50 years of age, if they are in adequate physical and mental condition to be interviewed, and if they are willing to participate in the study as confirmed by signed informed consent. Some of the new cases reported by the dialysis centers (documented in the logbook) are expected to meet not the eligibility criteria. It was expected that more than 70% of reported cases are eligible (eligibility rate). A response rate of about 80% of all eligible incident cases admitted in the respective dialysis unit (as documented in their logbook) was assumed for the sample size calculation.

Cases are invited to participate in the study by the treating physician at the dialysis center, who explains the objectives of the study as being "research into associations between chemicals and health". Care is taken that no reference is made to a suspected association between renal failure and analgesics. The physician provides the patient with the invitation letter, which is identical for cases and controls and which contains details on the interview process as well as examples of the questions asked in the interview. If the patient agrees to participate, his or her contact details are sent to the Data Management and Coordinating Center (DMCC: ZEG Berlin) that informs the interviewer staff to make an appointment. The interviewer then visits the patient at home after having made an appointment. No interview is done without a signed informed consent form.

All cases and controls were normally interviewed in person at their homes on the basis of a detailed and structured questionnaire. The same interviewer conducts all interviews for each case-control cluster (one case and up to four controls).

The interview provides information about exposure, relevant co-variables, and times of exposure (diary or calendar method), permitting the full use of the time-related variables for the statistical evaluation of time dependent data or pre-planned sub-groups (cf. analyses plan).

#### Controls

The study is designed to use population controls as reference group. Eligible controls are persons without ESRD aged less than 50 years matched individually for age (same 5-year age group as the case), sex and region of the respective case at entry in the dialysis program. Controls are eligible if they are willing to participate in the study, provide informed consent, and are in adequate physical and mental condition to be interviewed. Controls may have any medical conditions except ESRD to be eligible, because the controls should represent the population with regard to medical conditions. In equivocal circumstances (e.g. controls with kidney stones or other kidney diseases), controls will be considered separately in the analysis but will not be stopped to complete the interview.

Controls are recruited after the case interview is completed and the case's matching region is known. Four matched controls were tried to accrue per case. If one potential control refuses participation the next eligible person replaces him or her. The method of control identification varies depending on location. Whereas for larger cities listings of residents form the basis for recruitment, smaller villages are more suited for random route sampling and neighborhood or telephone contacts.

A response rate of 60% was assumed (after correction for non-eligible controls resulting from death, incorrect address, and other reasons that prevent to get into contact with the potential control person). Based on the experience from recent population-based case-control studies in Germany, this response rate is a realistic estimate.

### Non-participation, and non-response for cases and controls

There are various reasons for non-participation in the study or exclusion from the analysis for individuals who were considered initially eligible to participate in the study.

The dialysis centre for various reasons might inadvertently miss eligible cases or report cases that are not eligible. These are documented in the logbook and expressed in the "eligibility rate" on the basis of all new patients reported from each of the collaborating centers.

An identified case might become ineligible due to poor physical or mental condition, insurmountable language problems, and death after identification but before interview, dialysis outside the study period, or being outside the age limit. Some of these reasons also apply to ineligibility of population controls. The proportion ineligible cases for these and other reasons forms the "eligibility rate" (Number of eligible cases divided by the number of initially identified cases

Eligible cases or controls may refuse to sign the informed consent and decline participation, or refuse to complete the interview. This is called "non-response" in the context of this study (Number of included/interviewed cases divided by the number of eligible cases).

Finally, cases or controls will be excluded from the analysis due to the use of any phenacetin-containing analgesic in any dose during their lifetime.

### Exposure and co-variables

#### Analgesics

The main exposure variable is analgesic use. A lifetime history of analgesic use is obtained during the personal interview. Memory aids include a list of brand names of analgesics and an atlas with pictures of packages of all analgesics, both past and present. Participants are asked for the brand names, number of daily doses, start and stop of use, and the reasons/indications for use. The brand names were used to identify all analgesics whether prescription or over-the-counter (OTC), single-substance and combination analgesics with and without caffeine, phenacetin, or additional substances such as codeine and barbiturates. Information is also collected on switching analgesics and the reasons for switching, as well as on subjective symptoms of "dependence" and psychological or behavioral patterns.

The exposure definitions are similar to those of two recent studies in Germany (MURC study [9] and Pommer et al [10]), to facilitate comparisons with these studies that are the only available on analgesic use in Germany or Austria, particularly on OTC analgesics.

The cumulative lifetime use of all analgesics and of specific analgesic subgroups will be based on grams of the respective analgesic group (see below). The cumulative lifetime dose of analgesics will be stratified into tertiles to obtain the distribution in population controls, being called low, moderate, and heavy use. The Scientific Advisory Committee (SAC, see later) recommended the use of the top tertile of lifetime analgesic dose (grams) as the measure for heavy analgesic exposure in preference to the definition of "substantial use" as it was defined in the study protocol before revision. This decision was made after data from the population controls in the planned review (according to the first protocol) showed that the utilization of analgesics had changed considerably in the decade since "substantial use" [10] was defined:

- "Heavy users" are defined as persons whose use of analgesics is in the top tertile of the accumulated lifetime dose in grams. Alternatively, heavy use can also be defined in a similar way for shorter periods, i.e. not for lifetime but for any of 12-months periods or other units. In contrast to lifetime use this was defined as "peak use". However, the main analyses will be based on cumulated lifetime dose that will have priority for answering the main research question.

- "Non-users" are defined as never-users or short-term/irregular users of any analgesics (<1 dose of analgesic per month over all possible previous 12-month periods). Participants matching this definition are members of a unique, general reference group which will be identical across all comparisons.

Persons with recalled use of phenacetin-containing analgesics in the past will be excluded from the main analyses of the study – even if phenacetin use cannot be confirmed because of vague recall.

The coding of lifetime use of analgesics follows the discussion and agreement of the Advisory Committee with a classification into 13 categories (see Table 1).

Due to the low prevalence for some analgesic groups found in the analyses of the drug use in controls after year one of the study, two broader categories (sub-categories) of phenacetin-free analgesics were formed: MONOS (all monos; paracetamol; ASA; rest of monos) and COMBIS (all combis; combis with paracetamol; combis without paracetamol; combis with caffeine; combis without caffeine).

These categories of lifetime use of analgesics are not mutually exclusive. Pure groups of users of one type of analgesics are very rare and only available for very commonly used analgesics such as ASA. This distinguishes an observational study from a clinical trial and requires an analysis adapted to the prevalence in users as found in the database. The magnitude of usage (lifetime dose, peak dose, duration, dose per duration of use) will be examined by categorizing these variables into tertiles or other groupings depending on the number of users in each group.

#### Other variables

The following co-variables, evaluated as independent risk factors for either initiation or promotion of ESRD, will be considered as potential confounders or effect modifiers of a potential association between ESRD and analgesic use:

Age, sex, education, region, selected conditions (renal diseases, urinary tract infections, family history of renal diseases, repeated abdominal X-ray, psychological conditions), co-medication (rheumatic drugs, immunosuppressants, anti-cancer drugs, antibiotics, self reported dependence on any drugs), complaints (gastrointestinal, heart, vomiting, depression, anxiety, sleeplessness, fatigue, irritability, stress, eating problems, and various pain [joints, back pain, migraine, other headache, menstruation), and exposure to potentially nephrotoxic agents at the work site (such as heavy or other metals, special or general silicates, solvents, soldering/welding fumes).

### Sample size estimation

The sample size needed to address the three main research questions was estimated with the following assumptions: Alpha 5%, beta 20% (power 80%), case-control ratio 1:4, minimal detectable risk (odds ratio) 2.0, and revised prevalence of heavy analgesic use (lifetime dose in grams; top tertile of use in each of the analgesic groups; adapted to the more accurate estimates from the study population; old estimates (see ) based on empirical data of population controls of the SAN study from end 2003 (see table 2). The sample size of cases and controls in table 2 is based on prevalence estimates of heavy use in different analgesic groups and thereby provides an impression what comparisons are likely or unlikely to be finally analyzed.

We conclude from this table that our initial estimate of the required sample size (cf. 1^st ^study protocol in ) is still roughly valid. About 1000 cases of ESRD need to be identified in the co-operating dialysis centers to include 800 cases and 3200 controls in the final analysis which would result in sufficient power for the analysis of the main study questions and would additionally provide results for many but not all of the subgroups.

### Quality control & assurance measures

Quality control and assurance measures are implemented to ensure that the procedures and data are reasonably valid, and compatible within and among centers and between cases and controls.

The quality assurance measures were directed at the interview technique, the preparation of the field work, the conduct of the study, and finally at the plausibility of the database. The interview was standardized and the questionnaire was tested in a pilot phase for the clarity of its items. The interviewers were trained and re-trained and appropriate explanations were integrated into the interviewer training plan and guideline. A set of aids was introduced to reinforce the memory of study participants such as picture displays of analgesic packages, tables with lists of drugs for the study subject to read etc. to avoid recall bias. Training and retraining of all personnel involved in the study was done: of investigators, abstractors of medical records, interviewers, following a standardized training plan.

A detailed logbook was kept in each of the participating dialysis centers. This provides information on the eligibility or ineligibility of cases and controls, participation rates and the reasons for non-responses or non-participation, the status of recruitment and other details. Additionally, site visits were done, if required.

Incoming data were checked in the Data Management and Coordinating Centre Berlin for quality and comprehensiveness by a sophisticated quality assurance system and queries were made to the study center if there was doubt about the validity of the data or if there were missing data.

### Data management and analysis plan

#### Data management

The data were collected locally and thereafter transferred to the central Data Management and Coordination Centre (DMCC) Berlin according to clear time lines and defined responsibilities. The DMCC consists of ZEG Berlin (data management) and EPES Berlin (data analysis).

The data were entered into the database and thoroughly checked for errors and plausibility. The database will then be transferred into the "clean data set" that will contain the following groups of data:

1. Case/control variable ESRD (diagnostic categories, time variables as far as available)

2. Main exposure variables – use of analgesics (up to the time of respective index dates)

3. Co-variables:

- Co-morbidity: renal, circulatory, metabolic, psychiatric, and other conditions; headache and other painful conditions;

- Treatment history: types of treatment with potential relation to outcome and risk factors;

- Exposure to caffeine: coffee, tea, and caffeine-containing beverages;

- Job exposure: occupation & industrial branch; exposure in selected occupational categories; exposure to groups of certain chemicals/minerals;

- Other personal data: Age, sex, centre, health care contacts, socio-demographic markers

4. Factors potentially related to overuse of analgesics: psychological / vegetative / psychiatric conditions; behavioral factors (such as smoking, alcohol, physical activity).

Several databases will have to be established for different analyses which relate to the different index dates, different classifications of user groups of analgesics etc.

#### Analysis plan

In the final analysis, appropriate multivariate analyses will be done to assure that the risk estimates for ESRD and use of phenacetin-free analgesics are appropriately adjusted for confounding and effect modification. A detailed analysis plan was discussed at the meeting of the SAC in April 2004, and approved after revisions. Briefly, the "core analyses" will include:

- Information on selection such as included cases by regions/country, non-participation rate by case/control status, non-response rate by case/control status, reasons for non-participation and non-response by case/control status.

- Frequency distributions of exposure with analgesics by case/control status and by index dates such as ever vs. never use, lifetime dose (grams) in tertiles vs. non-use, duration of use (years) in tertiles vs. non-use, density of use (dose per duration of use).

- Logistic regression analyses of ESRD and analgesic exposure by index dates: Ever vs. never use, lifetime dose (grams) in tertiles vs. non-use, duration of use (years) in tertiles vs. non-use, density of use (dose / duration).

Several methodological issues will be addressed in the analyses:

One such issue relates to the precise definition and status of the index dates. Index date 1 is used only for description of the group of cases and controls, but not for the interpretation of the risk of nephropathy. The preferable index time for risk estimates is index 3. If the exposure data at index date 3 are insufficient, then SAC endorsed the use of index date 2.

The analgesic groups analyzed in the "core analyses" are not mutually exclusive. This means that the analysis of heavy lifetime use in one specific analgesic group is usually contaminated by use in other analgesic subgroups. It is unlikely to find sufficiently large "mutually exclusive groups of heavy analgesic use" will exist in the database. Moreover, it is unlikely that a satisfactory statistical approach for this problem will be found. The final decision on which method will be employed depends on the quantity of data available.

The inclusion of dosage of a specific analgesic subgroup (grams) as a continuous variable was discussed to provide a more comprehensive analysis of the dose-response effects in the "normal" user population. Such a variable might preferably be defined in broader categories of grams, showing not the increase of risk per one gram but per 10 or 50 or 100 or even 500 grams of lifetime dose.

An analysis of peak use might be appropriate if the data indicate a consistently increased risk in some of the analgesic subgroups. Peak use is defined as high use within a brief period of time (12 month) with only little or average use before and after this episode. This approach differs from the analyses of the cumulated lifetime dose, and it is not the focus to answer the main research question.

These and other data-driven analyses are suitable to test or confirm the biological plausibility of results. However, these analyses are hypothesis generating and will not be conducted to answer the planned main research questions.

The final analysis will face the problem of small numbers in many analgesic subgroups. The SAC strongly recommended reducing the number of adjustment variables to an absolute minimum. The appropriate statistical methods will be applied to select the most important confounders to get more stable risk estimates for small numbers. Further, the committee recommended not calculating or report adjusted odds ratios if any cell contains less than 10 subjects.

The findings must be interpreted with the consideration in mind that numerous analyses will be done to scrutinize the many available databases. Therefore it is statistically expected that some significant results will be found. This does not necessarily reflect a causal association because it may be a statistical artifact. The option of controlling for multiple testing in an observational study was discussed but not finally decided. It might be sufficient to interpret the results with great caution without formally applying further statistical procedures.

### Study management

The management of the study with regard to clinical/nephrological aspects, including recruitment of co-operating dialysis centers, is the responsibility of Prof. van der Woude (V. Medizinische Klinik- Nephrologie, Klinikum Mannheim, Medizinische Fakultät der Universität Heidelberg) for Germany, and of Prof. Graf (Abteilung für Nephrologie des Krankenhauses Rudolfstiftung in Wien) for Austria.

The management of the whole study with regard to epidemiological expertise, data management and analyses is the responsibility of Prof. Heinemann (Centre for Epidemiology & Health Research Berlin).

The three Principal Investigators mentioned above form the Managing Committee of the SAN study in Germany and Austria.

The German Kidney Foundation plays a key role in supporting the study, particularly with regard to co-operation with the three German Nephrological Associations (see acknowledgments) and thereby with the collaborating dialysis centers. The same role plays the Austrian Nephrological Association for this country.

Agreements on terms of reference of various partners in the study, on ownership of data, accessibility of the database, and publication policy are available on the website of the SAN study . This website also contains the current status of the fieldwork and other accessible data.

### Advisory committees

The scientific advisory committee (SAC) was jointly nominated by the Drug Authorities and the Industry. It consists of internationally acknowledged experts for different fields of expertise. The terms of reference of this committee and the names of its members are detailed in annex 1 and on the SAN website . The SAC also discussed and approved revisions of the protocol. It will finally approve the main analyses and the main publication prior to submission.

Delegates of the Nephrological Associations (three German and one Austrian Association) formed a Steering Committee to support the study. Two members of each Association of the two countries where the study is conducted have a seat in this advisory committee. The steering committee received monthly reports from the study. It can request additional information, and supports the study by identifying and resolving problems that occur within the study period. Details about Terms of Reference and names of members can be found on the SAN website .

### Communication

Periodic reports of study progress are made to the SAC, and the Steering Committee of the study.

Additionally, annual interim analyses for selected questions were submitted to the SAC for discussion and to get advice. Summaries of the annual reports were submitted to interest Drug Regulators only after approval by the SAC.

A publication policy was agreed with the collaborating study centers (see  for details).

The study will be analyzed and published in 2005/06.

## Discussion

Because confounding and bias can affect every observational study, potential forms of bias received special consideration in the design of the initial study protocol in 2000/2001. Several sources of bias, such as recall bias, selection bias, reverse causality (protopathic) bias, interviewer bias, and (residual) confounding have been addressed in critical reviews of previous case-control studies on the association between analgesics and kidney disease [5,6] and were of concern in the design of this case-control study.

Recall bias assumes a differential recollection of exposures between cases and controls. In order to minimize recall bias, cases and controls are interviewed in their homes to provide for a comparable interview situation. Interviewers are trained to conduct standardized interviews using the same material in cases and controls. Visual and other aids are used to facilitate memory. These include lists of the trade names of analgesics and other drugs which were or are on the market, pictures of packages of different analgesic brands etcetera. Drug use is ascertained according to the calendar method so that a lifetime history of analgesic use can be constructed for each study subject. Interviewers are trained to ask for beacon points in the patients' lives in order to facilitate memory of the time period of drug use.

Although differential recall often tends to lead to inflated risk estimates, it is also of concern in this study that patients with analgesic nephropathy may deny their previous analgesic intake that would bias the risk estimate for analgesics towards the null. Therefore, in the personal interview, questions about medical conditions for which analgesics may have been taken precede questions about analgesic drug use. If the patients report medical conditions for which analgesics may have been used but do not report exposure to analgesics the interviewers have been instructed to ask the patients again about drug use for the respective conditions.

Selection bias: Since it was assumed that cases of ESRD are equally identified among analgesic users and nonusers due to the severity of their medical condition, exposed subjects should not have a higher likelihood of being selected into the study. This could be a problem if cases were defined as patients with early renal disease and analgesic users had more serum creatinine measurements done and therefore a higher likelihood of being detected as a case. For the endpoint ESRD no selection bias due to differential selection of exposed cases is anticipated. Selecting cases with advanced disease has been criticized in previous case-control studies, since the etiologically relevant exposures are those that antedate the onset of disease and not those that antedate end-stage renal disease. To address this concern, several index dates are considered (see reverse causality bias).

The study uses a largely population-based approach (primary study base) by attempting to recruit all regional dialysis units located in these regions (usually federal states), enrolling all incident cases in these units into the study and by selecting population controls from the same regions where the case came from. The representativeness of the control group can be investigated by comparing characteristics in controls to those in the population using official statistics as a source.

A system of motivation and reminder is in place to assure adequate response rates in both cases and controls. In order to avoid non-participation of cases with analgesic nephropathy, the information on the type of question in the study information is subtle and avoids mentioning of analgesics.

Reverse causality (protopathic) bias is also of concern in this study. Chronic renal disease may lead to symptoms or physical changes (for example backache, headache, malaise, intercurrent infections, fever etc), which in turn may lead the patient to use analgesics. Exposure to analgesics in cases of ESRD may therefore reflect exposure which truly started before the chronic renal disease process or which was a consequence of the disease process.

It is very difficult to address this type of bias in a case-control study. To minimize reverse causality bias, several index dates are considered in the statistical analyses. One index date to be considered is the date the renal disease was first suspected/diagnosed (taken from the interview where the patient is asked when the doctor first mentioned an increased serum creatinine level). However, this date may already represent an advanced stage of the disease, since chronic renal disease commonly has an insidious onset and therefore is often not diagnosed in the early stages. Therefore, two other index dates are considered which are based on fixed lag-times of 5 and 10 years. This approach is commonly used in cancer epidemiology and avoids bias by (differential) detection of the disease. Discrepancies in results between index date 5 and 10 years could be indicative of protopathic bias (cf. discussion). Protopathic bias cannot be ruled out if the risk estimate for ESRD in analgesic users is increased with use of the index date with a lag time of 5 but not with a lag time of 10 years., According to recommendations of the SAC, the index date with a lag time of 5 or 10 years is given priority in the analysis to avoid protopathic bias (see above).

Interviewer bias: Although it will not be possible to blind interviewers to the case or control status, interviewers are blinded to the main study hypothesis. The interview does not only ask about kidney disease, but also about a broad range of health outcomes. Furthermore, interviewers acquire information not only about the use of analgesics, but also about exposure to many other drugs as well as exposure to many chemicals at the work place.

Confounding: Previous studies were criticized for confounding by chronic phenacetin use. In this study, chronic phenacetin use will be identified by means of a database that lists the names of all analgesics that contained phenacetin in the past and the respective time periods in which these analgesics contained phenacetin (since the composition of analgesics was often changing in Germany, but the trade name of the drug remained the same). With this database and the calendar method in the interview it will be possible to identify patients with significant phenacetin use and exclude them from testing the main study hypotheses. This study should provide a better opportunity to study the association between phenacetin-free analgesics and chronic renal disease than previous case-control studies, since only patients below age 50 are included. Also, many of the earlier studies were conducted more than 10 years ago when phenacetin use had not been banned long enough to enroll a large enough number of patients without significant phenacetin use. Now, more than 10 years later, this will be more likely possible.

Due to methodological limitations of previous case-control and cohort studies the definition of significant phenacetin use is not straightforward. The cumulative amount of phenacetin that carries an increased risk of chronic kidney disease (and therefore which patients with phenacetin use in the past are to be excluded) is not clear. If the threshold value for significant phenacetin use defined is too high, residual confounding by phenacetin use may lead to an inflated risk estimate for non-phenacetin containing analgesics. Previous studies have shown high risk estimates for ≥ 1 kg cumulative dose of phenacetin: McCredie (1988)[11]: OR = 19 (10–37); Pommer (1989)[10]: OR = 9 (2–39) and also for regular use of phenacetin: Morlans (1990)[12]: OR = 19 (2–157); Sandler (1989)[13]: OR = 5 (1.7–14.9). Some studies have also suggested an increased risk for smaller doses of phenacetin. McCredie observed an odds ratio of 15 (8–28) for ≥ 0.1 kg of phenacetin (compared with < 0.1 kg of phenacetin) and in the study by Pommer [10], a significant increase in risk was observed for cumulative doses of phenacetin of 100–499 g of phenacetin (OR = 1.99 (1.14–3.48)) and 500–999 g (OR = 2.58 (1.19–5.59). No increased risk was observed in this study for cumulative doses of phenacetin of less than 100 g. Sandler[13] also observed an increased odds ratio for lower doses of phenacetin (OR = 1.92 (1.06–3.49) for weekly use of phenacetin defined as use at least once a week for at least one year). To avoid confounding by residual phenacetin use, we will exclude all cases and controls with any detectable phenacetin use from the test of the main study hypotheses.

It is also very important to ascertain information on medical conditions which can independently affect kidney function and lead to an increased use of analgesics such as rheumatic disorders, diabetes, hypertension, kidney stones etc. This study carefully collects information on these conditions and their date of occurrence in the medical documentation form and in the interview in order to be able to control for these conditions in the statistical analyses.

## Competing interests

Investigators, drug regulators and the pharmaceutical companies in a common approach initiated this study. The investigators and the SAC exclusively designed the study with some critical remarks from drug regulators and industry. The SAN study is scientifically independent and governed by an independent Advisory Committee. A group of pharmaceutical companies (Boehringer-Ingelheim Pharma GmbH&Co.KG; Dr. Mann Pharma; Whitehall-Much GmbH; Berlin-Chemie AG; Dr. R. Pfleger GmbH; Roche Consumer Health) provided an unconditional grant to cover the costs of the preparatory meetings, the conduction of the study, and the meetings of the advisory committees. The investigators are accountable to the SAC in all scientific matters. None of the investigators have any financial relationship with the group of manufacturers of analgesics.

## Authors' contributions

**LAJH**: Responsible for the epidemiological study design and involved in writing of the paper. **EG**: Contributed to the study design and to writing & revising the paper. **ML**: Contributed to the study design particularly data management, analysis plan, and revision of the paper. **FvdW**: Responsible for the nephrological issues of the study protocol, the collaboration of German nephrological societies and dialysis centres and contributed to writing/revising of the paper. **HG**: Responsible for the nephrological issues, the collaboration of Austrian nephrological society and dialysis centres, and contributed to writing/revising of the paper.

## Appendix 1

### The Scientific Advisory Committee (SAC)

Prof. A. R. Feinstein, Dept. Epidemiology, Yale University, Medical School, New Haven, USA: (Chairman until end 2001, died)

Prof. K.-M. Koch, Abt. Nephrologie, Medizinische Hochschule Hannover, Hannover, Deutschland: (Chairman from 2002)

Prof. S. Shapiro, Div. Epidemiology, School of Public Health, Columbia University, New York, USA (from 2002)

Prof. P. Bauer, Institute for Medical Statistics, University of Vienna, Vienna, Austria

Prof. E. Delzell, Occupational Epidemiology, Scholl of Medicine, University Birmingham, USA

Dr. G. Curhan, Channing Laboratory, Harvard University, Boston, USA (until 2004)

Prof. J.M. Fox, University of Saarland, Germany

Prof. P. Michielsen, University of Leuven, Belgium

Prof. S. Suissa, Pharmacoepidemiology Research Unit, Div. Clinical Epidemiology, McGill University, Montreal, Canada

Dr. B. Kasiske, Internal Medicine & Nephrology, Hennepin County Medical Center, University Minneapolis, Minneapolis, USA (from 2002)

Prof. M. Mihatsch, Institute for Pathology, University Basel, Switzerland

### The Steering Committee (SC)

Prof. Dr. H. Holzer, Nephrologie, Universitätsklinik, Graz, Austria

Prof. Dr. W. H. Hörl, Medizinische Klinik III am Allgemeinen Krankenhaus der Stadt Wien, Austria

Prim. Doz. Dr. H.K. Stummvoll, Krankenhaus der Elisabethinen, Linz, Austria

Prof. Dr. Florian Lang, Generalsekretär der Gesellschaft für Nephrologie, Tübingen, Germany

Prof. Dr. D. Schlöndorff, Medizinische Poliklinik Klinikum Innenstadt, München, Germany

Prof. Dr. T. Risler, Leiter der Sektion Nephrologie, Medizinische Klinik III, Tübingen, Germany

Prof. Dr. W. Boesken, II. Medizinische Abt., Krankenhaus der Barmherzigen Brüder, Trier, Germany

Prof. Dr. G. Schultze, Dialyse Institut Villingen-Schwenningen, Germany

### Study coordination

Germany: Dr. A. Busauschina, Dr. P. Schnülle (Klinikum Heidelberg-Mannheim, 5. Med. Klinik – Nephrologie, Mannheim, Germany

Austria: K. Stadler (2001/2002), G. Schneidewind (from 2002)

### International coordinating and data management center

Organization of field work, quality management, and data management: A. Assmann, Dr. S. Möhner, Dr. T. DoMinh (ZEG Berlin),

Data analyses: D. Kuehl-Habich (EPES Berlin)

Advisor for Clinical Pharmacology of analgesics: Dr. K. Farker, Institute for Clinical Pharmacology, Jena, Germany

### Statistical advisory group

Prof. Dr. S. Suissa, McGill University Montreal, Canada

Prof. Dr. P. Bauer, University of Vienna, Austria

Prof. Dr. J. Röhmel, German Drug Authority, Bundesinstitut für Arzneimittel und Medizinprodukte, BfArM, Bonn, Germany

### Collaborators in dialysis centers

The collaborators in the 170 German and Austrian dialysis centers are listed in the study website .

## Pre-publication history

The pre-publication history for this paper can be accessed here:



## Figures and Tables

**Figure 1:**
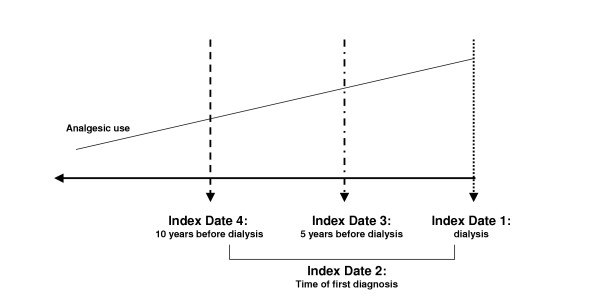
Index dates and relationship with putative analgesic use in the SAN case-control study.

**Table 1:** Categories of lifetime use of analgesics

**Category of analgesics**	**Description**
**1. Phenacetin-free analgesics**	All analgesics not containing phenacetin
**2. Phenacetin-containing analgesics**	Analgesics containing phenacetin
**3. Mono analgesics (MONOS); phenacetin-free** One analgesic compound, no non-analgesic active agents (except vitamins)	
	3.1 Paracetamol (acetaminophen)
	3.2 ASA
	3.3 Ibuprofen
	3.4 Metamizol, Diclofenac, Propyphenazon, Indometacin, Aminophenazon, Phenylbutazon)
**4. Combination analgesics (COMBIS); phenacetin-free** Combination of more than one active analgesic agent – or – one or more analgesic active agent with one or more non-analgesic agents	
	4.1 Paracetamol + caffeine (Paracetamol and caffeine, no other analgesic or non-analgesic agent)
	4.2 Paracetamol + ASA (Combination of paracetamol and ASA, no other analgesic or non-analgesic agent, no caffeine, no codeine, no barbiturates
	4.3 Paracetamol + ASA + Caffeine (Combination of paracetamol and ASA and caffeine, no other analgesic or non-analgesic agent)
	4.4 Other monos + Caffeine (One other analgesic agent – except phenacetin or paracetamol with caffeine, no additional non-analgesic agents
	4.5 Other combis without caffeine (Two or more analgesic agents – except combination paracetamol + ASA with or without non-analgesic agents, or one analgesic agent with one or more non-analgesic agents, but no caffeine, no codeine, no barbiturates.
	4.6 Other combis + caffeine (ASA or paracetamol and/or other analgesic agents and caffeine, no phenacetin, no codeine, no barbiturates)
	4.7 Combis with codeine and/or barbiturates (One or more analgesic agents and codeine and/or barbiturates, independent of other agents, i.e. even with additional caffeine, but no phenacetin.

**Table 2:** Number of cases and controls needed to detect a 2-fold risk (alpha 5%, 1-beta = 80%). Comparison of heavy analgesic use (top tertile in grams) vs. non use in the respective categories of analgesics. Important: the subcategories are not mutually exclusive and cannot be added up to 100%.

	Prevalence in controls (%)	Nb of cases	Nb of controls
All analgesics (phenacetin-free)	22.8	104	416
Phenacetin-containing	2.5	616	2464
***MONOS***			
Paracetamol	5.3	308	1232
ASA	15.7	129	516
Ibuprofen	2.1	728	2912
Other monos	5.0	324	1296
***COMBIS***			
Paracetamol+caffeine	0.1	14 670	58 680
Paracetamol+ASA	0.9	1657	6628
Paracet+ASA+caffeine	4.1	388	1552
Others+caffeine	0.6	2470	9880
Other combis without caffeine	3.5	449	1800
Other combis+caffeine	3.3	475	1900
Combis with codeine or barbiturates	1.9	654	2616

## References

[B1] Dubach UC, Levy PS, Minder F (1968). Epidemiological study of analgesic intake and its relationship to urinary tract disorders in Switzerland. Helv Med Acta.

[B2] Grimlund K, ktryckeriet Bo (1963). Phenacetin and renal damage at a Swedish factory. Kungl.

[B3] Delzell E, Shapiro S (1998). A review of epidemiologic studies of nonnarcotic analgesics and chronic renal disease. Medicine.

[B4] McLaughlin JK, Lipworth L, Wong-Ho C, Blot WJ (1998). Analgesic use and chronic renal failure: A critical review of the epidemiologic literature. Kidney International.

[B5] Feinstein AR, Heinemann LAJ, Curhan GC, Delzell E, DeSchepper PJ, Fox JM, Graf H, Luft FC, Michielsen P, Mihatsch MJ, Suissa S, Van der Woude F, Willich S, (ad-hoc review committee) (2000). The relationship between non-phenacetin combined analegesics and nephropathy: A review. Kidney Internat.

[B6] Hersh EV, Moore PA, Ross GL (2000). Over-the-counter analgesics and antipyretics: a critical assessment. Clin Ther.

[B7] Feinstein AR, Heinemann LAJ, Dalessio D, Fox JM, Goldstein J, Haag G, Ladewig D, O'Brien CP (2000). Do Caffeine-containing analgesics promote dependence? A review and evaluation. Clin Pharmacol Ther.

[B8] Fox JM, Siebers U (2003). Caffeine as a promotor of analgesic-associated nephropathy – where is the evidence?. Fundamental & Clinical Pharmacology.

[B9] Greiser E, Molzahn M, eds (1998). Multizentrische Nieren- und Urothel-Carcinom-Studie (Abschlußbericht). Schriftenreihe der Bundesanstalt für Arbeitsschutz und Arbeitsmedizin Fb 780.

[B10] Pommer W, Bronder E, Greiser E, Helmert U, Jesdinsky HJ (1989). Regular analgesic intake and the risk of end-stage renal failure. Am J Nephrol.

[B11] McCredie M, Stewart JH, Mahony JF (1982). Is phenacetin responsible for analgesic nephropathy in New South Wales. Clin Nephrol.

[B12] Morlans M, LaPorte J, Vidal X, Cabeza D, Stolley PD (1990). End-stage renal disease and non-narcotic analgesics: a case-control study. Br J Clin Pharmacol.

[B13] Sandler DP, Smith JC, Weinberg CR, Buckalew VM, Dennis VW (1989). Analgesic use and chronic renal disease. N Engl J Med.

